# Case Report: Reversal of Long-Standing Refractory Diffuse Non-Scarring Alopecia Due to Systemic Lupus Erythematosus Following Treatment With Tofacitinib

**DOI:** 10.3389/fimmu.2021.654376

**Published:** 2021-04-14

**Authors:** Yu-Lan Chen, Li-Xiong Liu, Qin Huang, Xue-Ying Li, Xiao-Ping Hong, Dong-Zhou Liu

**Affiliations:** Department of Rheumatology and Immunology, Shenzhen People’s Hospital (The First Affiliated Hospital of Southern University of Science and Technology), The Second Clinical Medical College of Jinan University, Shenzhen, China

**Keywords:** Janus kinase inhibitor, tofacitinib, diffuse non-scarring alopecia, hair regrowth, systemic lupus erythematosus

## Abstract

The Janus kinases (JAKs) are intracellular tyrosine kinases involved in a broad variety of inflammatory cascades participating in the pathogenesis of systemic lupus erythematosus (SLE). Diffuse non-scarring alopecia is one of the most frequent cutaneous manifestations in SLE, resulting in devastating psychosocial consequences. Although recent studies have shown promising outcomes of the JAK inhibitors in SLE treatment, the efficacy of tofacitinib in diffuse non-scarring alopecia due to SLE has never been reported. Here we present a 29-year-old SLE patient with a 10-year history of refractory severe diffuse non-scarring alopecia who experienced dramatic hair regrowth with tofacitinib. Furthermore, we have made a systematic review regarding the potential effectiveness of tofacitinib in systemic and cutaneous lupus erythematosus. To the best of our knowledge, this is the first case study depicting an SLE patient with refractory alopecia who experienced impressive hair regrowth with the JAK1/3 inhibitor tofacitinib therapy, which contributes to expanding the field of possible uses of tofacitinib in SLE patients with difficult-to-treat cutaneous involvement, including severe alopecia.

## Introduction

Alopecia is one of the most frequent cutaneous manifestations in systemic lupus erythematosus (SLE) (1), potentially reflecting SLE disease activity (2). It often manifests as a diffuse non-scarring form of hair loss, sometimes even involving more than 50% of the scalp surface area. Although alopecia is not life-threatening, it can be associated with devastating psychosocial consequences, resulting in depression, anxiety, and poor quality of life (3).

The Janus kinases (JAKs) are intracellular tyrosine kinases involved in a broad variety of inflammatory cascades participating in the pathogenesis of SLE (4). Tofacitinib is a JAK1/3 inhibitor licensed for the treatment of moderate to severe active rheumatoid arthritis, psoriatic arthritis and ulcerative colitis (5). It has been shown that tofacitinib ameliorates both clinical and histological features of lupus-associated skin inflammation in murine lupus (6). A recent study including 10 patients with SLE demonstrated that tofacitinib could rapidly improve the signs and symptoms of arthritis and partially ameliorate skin rash (7). However, the efficacy of JAK1/3 inhibitors in diffuse non-scarring alopecia due to SLE has never been reported. Here, we describe an SLE patient with long-standing severe diffuse non-scarring alopecia who experienced dramatic hair regrowth with tofacitinib.

## Case Presentation

A 29-year-old Chinese woman presented with a 10-year history of diffuse non-scarring alopecia and recurrent rash. At the age of 12, she was diagnosed with SLE due to severe generalized rash with pruritus, alopecia, hypocomplementaemia with complement 3 to be 0.53 g/L (reference range 0.80 - 1.81 g/L) and complement 4 to be 0.11 g/L (reference range 0.15 - 0.57 g/L), an elevated titre of anti-nuclear antibodies (ANA, 1:1000, speckled immunofluorescent pattern; reference range < 1:100) and positive anti-double-stranded (ds) DNA antibodies determined by indirect immunofluorescence. Her anti-Sjögren’s syndrome antigen A (SSA) antibodies were also positive by immunoblotting and the level of serum immunoglobulin G was 18.66 g/L (reference range 8.0 - 18.0 g/L) with normal complete blood cell count and 24-hour urine protein. SLE disease activity was stable after remission for 7 years with low-dose methylprednisolone (4 mg/day) and hydroxychloroquine (200 mg/day) for maintenance therapy.

At the ages of 19, 23, and 24, she had three SLE flares with rash and hair loss progressing over several days to nearly her entire scalp. Methylprednisolone (40 mg/day) combined with cyclosporine (75 mg twice a day) following high dose pulsed methylprednisolone at 250 mg/day for 3 days significantly relieved her symptoms, with methylprednisolone (4 mg/day) and hydroxychloroquine (200 mg/day) for maintenance therapy. However, at the age of 25, her diffuse non-scarring alopecia relapsed again. The severe alopecia was persistent for the next 4 years without any therapeutic benefit from the previous regimen mentioned above (methylprednisolone at a dose of 40 mg/day combined with cyclosporine after steroid pulse therapy), methotrexate, mycophenolate mofetil, hydroxychloroquine, or oral or topical tacrolimus. Nevertheless, she refused to undergo scalp skin biopsy. Moreover, her rash had also recurred intermittently during this period. Two weeks before admission, methylprednisolone (24 mg/day) was administered in a local hospital due to the recurrence of a generalized rash with pruritus, but it had a negligible effect.

**Figure 1:**
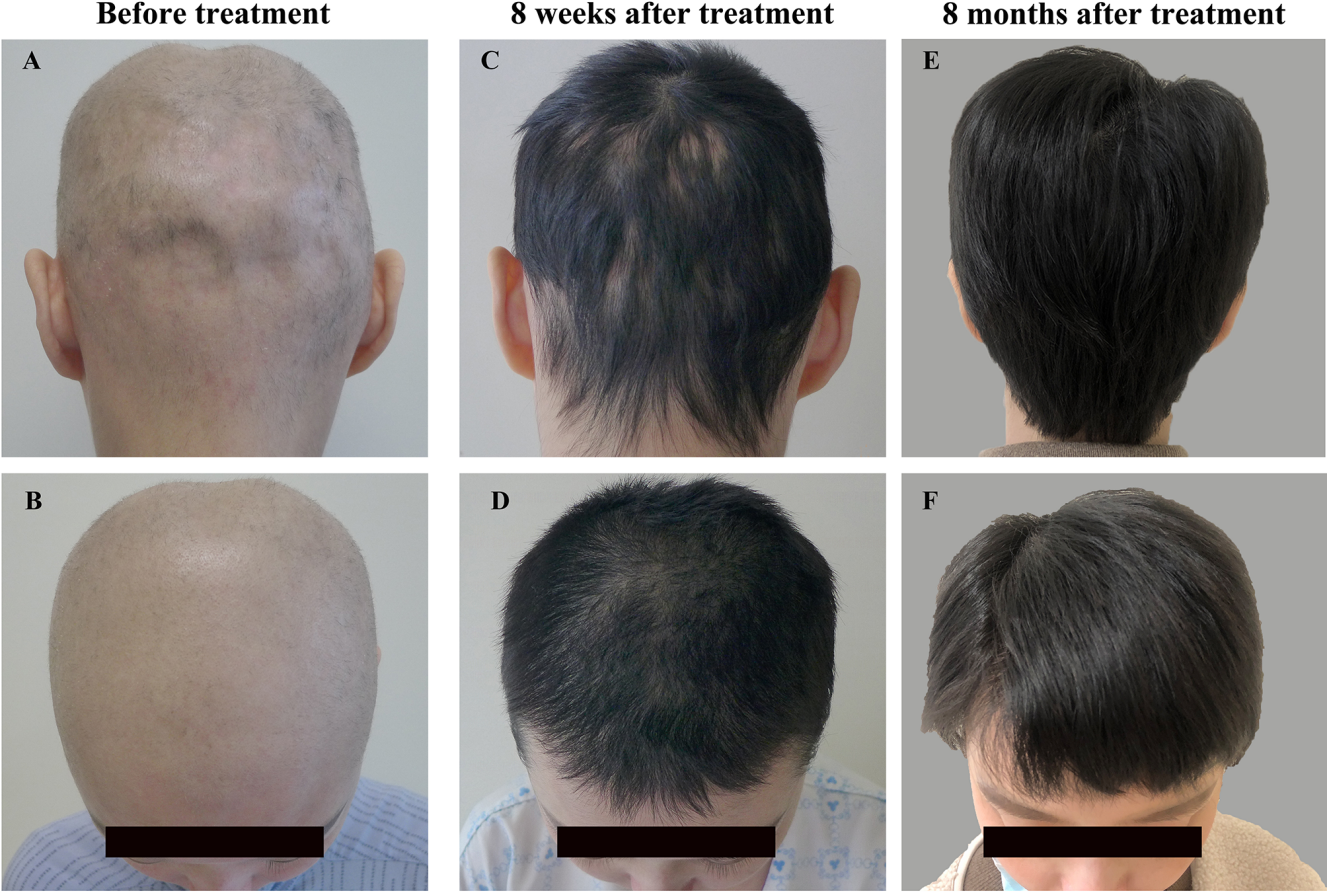
Changes in scalp hair on admission **(A, B)**, 8 weeks **(C, D)** and 8 months after tofacitinib therapy **(E, F)**.

Physical examination on admission revealed generalized rash with skin scratches and diffuse non-scarring alopecia involving nearly the entire scalp (**Figures 1A, B**). Laboratory findings revealed positive ANA (1:320, speckled immunofluorescent pattern; reference range < 1:100) with negative anti-dsDNA antibodies by indirect immunofluorescence and normal levels of complement components, as well as normal complete blood cell count and 24-hour urine protein. Owing to the reported promising outcomes of the JAK1/3 inhibitor tofacitinib for cutaneous involvement in SLE (7) and alopecia areata (8), tofacitinib (5 mg twice a day) was initiated along with hydroxychloroquine (200 mg twice a day), and methylprednisolone was gradually tapered. Surprisingly, prominent hair regrowth on the scalp was observed after 4 weeks, without any rash. At 8 weeks, the methylprednisolone dose was reduced to 8 mg/day with no relapse of alopecia or rash (**Figures 1C, D**).

At 7 months, she had her hair cut short because of her wedding. The tofacitinib dose was also tapered to 5 mg/day due to her plan to quit tofacitinib therapy and become pregnant. At the time of writing this report (8 months after tofacitinib therapy was initiated), the patient was being treated with 5 mg/day tofacitinib combined with 4 mg/day methylprednisolone and 200 mg twice a day hydroxychloroquine, with stable hair regrowth (**Figures 1E, F**). The timeline of the disease and treatment is shown in **Table 1**. Tofacitinib has been well tolerated, and no adverse events, including infection, have been observed in the patient up until the time of this report.

**Table 1:** Timeline of the disease and treatment.

Year of age	Symptoms/diagnosis	Treatment	Outcome
12	Rash with pruritus, alopecia, hypocomplementaemia, positive ANA and anti-dsDNA antibodies (a diagnosis of SLE was made)	mPSL (40 mg/day) and HCQ (200 mg twice a day) as initial therapy, with mPSL (4 mg/day) and HCQ (200 mg/day) for maintenance therapy	Improved and stable for 7 years
19, 23, 24	Rash and hair loss progressing over several days to nearly the entire scalp	mPSL (40 mg/day) and cyclosporine (75 mg twice a day) following pulsed mPSL at 250 mg/day for 3 days, with mPSL (4 mg/day) and HCQ (200 mg/day) for maintenance therapy	Improved
25	Hair loss involving nearly the entire scalp	mPSL (40 mg/day) and cyclosporine (75 mg twice a day) following pulsed mPSL at 250 mg/day for 3 days; methotrexate, mycophenolate mofetil, HCQ, or oral or topical tacrolimus	Alopecia was persistent and rash had recurred intermittently during the next 4 years
29	Rash with pruritus and hair loss involving nearly the entire scalp; positive ANA, normal levels of complement components, anti-dsDNA antibodies, complete blood cell count and 24-hour urine protein.	mPSL (24 mg/day) gradually tapered to 4 mg/day, tofacitinib (5 mg twice a day, tapered to 5 mg/day at 7 months), and HCQ (200 mg twice a day)	Improved

ANA, anti-nuclear antibodies; anti-dsDNA, anti-double-stranded DNA; SLE, systemic lupus erythematosus; mPSL, methylprednisolone; HCQ, hydroxychloroquine.

## Discussion

In this case study, although there was no other systemic involvement and current lupus immunologic evidence was almost absent except for positive ANA, a definitive diagnosis of SLE can be made in the patient due to her recurrent generalized rash, alopecia and hypocomplementaemia, as well as a high titre of ANA and positive anti-dsDNA antibodies. To the best of our knowledge, we describe for the first time, an SLE patient with long-standing refractory diffuse non-scarring alopecia who experienced dramatic hair regrowth with the JAK1/3 inhibitor tofacitinib, which contributes to expanding the field of possible uses of tofacitinib in SLE patients with difficult-to-treat cutaneous involvement, including severe alopecia.

The JAK-signal transducer and activator of transcription (STAT) pathway is regarded as a central communication node for the immune system that promotes many SLE associated pathogenic proinflammatory cytokines and chemokines, including interferons (IFNs), interleukin-6 (IL-6), IL-12 and IL-23 (9). The dysregulation of type I and II IFNs is a key signature associated with SLE, which provides evidence for using JAK inhibitors in patients with SLE (10). The pathogenesis of alopecia in SLE is still not clear. Previous studies have revealed that alopecia areata is driven by cytotoxic T lymphocytes and can be reversed by JAK inhibition through eliminating the IFN signature and subsequently inhibiting the downstream signaling pathway (11). Notably, according to the evidence mentioned above, a definitive diagnosis of SLE was made rather than idiopathic alopecia areata in this patient. Intriguingly, the severe diffuse non-scarring alopecia of our patient in whom standard treatments including pulse steroids and other immunosuppressive therapy (methotrexate, mycophenolate mofetil, hydroxychloroquine, or oral or topical tacrolimus) were not effective was successfully reversed by the JAK1/3 inhibitor tofacitinib. Therefore, it is reasonable to speculate that the IFN-JAK-STAT pathway may be involved in the pathogenesis of alopecia in SLE.

Tofacitinib is the first JAK inhibitor approved for RA, and recent studies have shown promising outcomes of tofacitinib in SLE treatment. *In vitro* and *in vivo* evidence regarding the potential effectiveness of tofacitinib in SLE and cutaneous lupus erythematosus is summarized in **Table 2**. Zhou et al. found that targeting JAK/STAT signaling by tofacitinib effectively controlled the fate of CD8^+^CD103^+^ tissue-resident memory T cells in lupus nephritis, contributing to reduced renal inflammation and damage in MRL/lpr mice (12). Tofacitinib challenge has been revealed to be effective in reducing the levels of anti-dsDNA antibodies and proteinuria, as well as in ameliorating nephritis in mouse models (13, 14). Moreover, lupus-associated skin inflammation could be significantly ameliorated by tofacitinib in murine lupus (6). In addition, Yamamoto et al. reported that tofacitinib decreased anti-dsDNA levels in one patient with inactive SLE complicated by rheumatoid arthritis (16). Intriguingly, a recent small cohort study demonstrated that tofacitinib could rapidly improve the signs and symptoms of arthritis and partially ameliorate skin rash in patients with SLE (7). Notably, our SLE patient presented with long-standing refractory alopecia and recurrent rash in whom there was negligible therapeutic benefit from standard treatments. Therefore, tofacitinib was initiated in the patient due to its reported promising outcomes for skin involvement in SLE (6, 7) and alopecia areata (8). Our case study further suggests that the JAK1/3 inhibitor tofacitinib may be a therapeutic option for patients with SLE, including individuals with difficult-to-treat cutaneous involvement in whom standard treatments are ineffective. Importantly, tofacitinib is now being investigated in SLE patients in a phase II clinical trial (NCT03288324).

**Table 2:** The effect of tofacitinib in systemic and cutaneous lupus erythematosus previously reported.

Studies	References	Year of publication	Case/case series/clinical trial	Results
**Cells/Animal**	(12)	2020	–	Improved kidney function in MRL/lpr mice
	(13)	2017	–	Reduced anti-dsDNA levels, decreased proteinuria, and ameliorated nephritis in NZB/NZW F1 lupus-prone mice
	(14)	2016	–	Reduced proteinuria and inflammatory cytokines, and ameliorated renal inflammation in NZB/NZWF1 lupus-prone mice
	(6)	2016	–	Ameliorated nephritis and skin inflammation, decreased ANA and anti-dsDNA levels, and improved vascular function in MRL/lpr lupus-prone mice
**Human**	(7)	2019	Case series (10 patients with SLE)	Rapidly ameliorated arthritis and partially improved skin rash
	(15)	2017	Case series (two patients with familial chilblain lupus)	Reduced discomfort and pain in the fingers
	(16)	2015	Case	Decreased anti-dsDNA titres to normal range in a patient with SLE and rheumatoid arthritis
	–	–	Phase II clinical trial NCT03288324	Ongoing

ANA, anti-nuclear antibodies; anti-dsDNA, anti-double-stranded DNA; SLE, systemic lupus erythematosus.

Other JAK inhibitors have shown the potential effectiveness in SLE and cutaneous lupus erythematosus. Evidence from a phase II clinical trial showed positive results with the JAK 1/2 inhibitor baricitinib in SLE, particularly at a dose of 4 mg/day (17). Fornaro et al. described one SLE patient with refractory papulosquamous subacute lesions who was successfully treated with baricitinib (18). Of note, one recent case report showed that a patient with SLE experienced substantial improvement in diffuse non-scarring alopecia following JAK1/2 inhibitor baricitinib therapy (19). Additionally, anti-extractable nuclear antigen (ENA) and anti-dsDNA antibody production in SLE patients could be abrogated in the presence of another JAK1/2 inhibitor, ruxolitinib (20). Moreover, Wenzel et al. reported a patient with chilblain lupus erythematosus that was successfully controlled by ruxolitinib therapy (21).

There is a paucity of evidence regarding the effect of JAK inhibitors on alopecia due to SLE. One recent case of diffuse non-scarring alopecia due to SLE successfully treated with the JAK1/2 inhibitor baricitinib therapy has been reported (19). The main limitation of the current study is that this is a case report. However, we have performed a systematic review, and to the best of our knowledge, this is the first case study depicting an SLE patient with refractory alopecia who experienced impressive hair regrowth with the JAK1/3 inhibitor tofacitinib, which exerted a profoundly positive effect on her quality of life. Therefore, our case adds to the small body of existing evidence on the promising outcomes of JAK inhibitors in alopecia caused by SLE. The efficacy and safety of tofacitinib in patients with SLE should be further defined by randomized controlled trials.

## Concluding Remarks

We describe the first case of an SLE patient with difficult-to-treat diffuse non-scarring alopecia who experienced remarkable hair regrowth with tofacitinib therapy, which may constitute a promising therapeutic option in SLE patients, especially in those in whom standard treatments are ineffective.

## Data Availability Statement

The original contributions presented in the study are included in the article/supplementary material. Further inquiries can be directed to the corresponding authors.

## Ethics Statement

The studies involving human participants were reviewed and approved by The Medical Ethics Committee of Shenzhen People’s Hospital (approval number: LL-KT-2018358). The patients/participants provided their written informed consent to participate in this study. Written informed consent was obtained from the individual(s) for the publication of any potentially identifiable images or data included in this article.

## Author Contributions

All authors contributed to the final manuscript. Y-LC analyzed documents and drafted the manuscript. Corresponding authors D-ZL and X-PH read and revised the manuscript. L-XL participated in drafting the manuscript. QH and X-YL collected the clinical data. All authors contributed to the article and approved the submitted version.

## Funding

Shenzhen Science and Technology Innovation Program (grant number JCYJ20190807144418845); Sanming Project of Medicine in Shenzhen (grant number SYJY201901); National Natural Science Foundation of China (grant number 81971464); and National Key Research and Development Program of China (grant number 2019YFC0840603).

## Conflict of Interest

The authors declare that the research was conducted in the absence of any commercial or financial relationships that could be construed as a potential conflict of interest.
